# An authoritative algorithm most appropriate for the prediction of pulmonary embolism in patients with AECOPD

**DOI:** 10.1186/s12931-020-01483-0

**Published:** 2020-08-18

**Authors:** Wei Xiong, He Du, Mei Xu, Wei Ding, Jinyuan Sun, Fengfeng Han, Xuejun Guo

**Affiliations:** 1 Department of Respiratory Medicine, Xinhua Hospital Affiliated to Shanghai Jiaotong University School of Medicine, No. 1665, Kongjiang Road, Yangpu District, Shanghai, 200092 China; 2 Department of Oncology, Shanghai Pulmonary Hospital Affiliated to Tongji University School of Medicine, Shanghai, China; 3 Department of General Practice, North Bund Community Health Center, Hongkou District, Shanghai, China; 4 Department of Pulmonary and Critical Care Medicine, Punan Hospital, Pudong New District, Shanghai, China

**Keywords:** AECOPD, Pulmonary embolism, Prediction, Algorithm

## Abstract

**Background:**

Contemporarily authoritative algorithms for the prediction of acute pulmonary embolism (PE) comprise the Standard algorithm, the Age-adjusted algorithm, the YEARS algorithm, the PERC algorithm, and the PEGeD algorithm. To date, little is known with respect to which algorithm is most appropriate for the PE prediction in patients with acute exacerbation of chronic obstructive pulmonary disease (AECOPD).

**Methods:**

The patients with AECOPD who underwent the confirmed chest imaging investigations of PE due to the likelihood of PE predicted by the Standard algorithm were retrospectively reviewed. The patients were reassessed by the other four algorithms to reveal which algorithm had the best diagnostic accuracy for the likelihood prediction of PE for patients with AECOPD.

**Results:**

The results showed that the PEGeD algorithm(88.6, 80.7, 50.4, 97.0%, 4.591, 0.141, 0.693, 82.1%) performed better overall in the sensitivity, specificity, positive predictive value, negative predictive value, positive likelihood ratio, negative likelihood ratio, Youden index, and diagnostic accuracy, in comparison with the Age-adjusted algorithm (78.6, 74.1, 40.1, 94.0%, 3.034, 0.289, 0.527, 74.9%), the YEARS algorithm (71.4, 76.6, 40.3, 92.4%, 3.051, 0.373, 0.480,75.6%), the PERC algorithm (98.6, 1.6, 18.2, 83.3%, 1.002, 0.875, 0.002, 19.2%). The difference of number of patients who were necessary to undergo chest imaging examinations and missed diagnoses resulted from each algorithm between the PEGeD algorithm and the Standard algorithm, the Age-adjusted algorithm, the YEARS algorithm, as well as the PERC algorithm were [− 789 (− 68.1%), N/A], [− 42 (− 3.6%),-21 (− 1.8%)], [− 3 (− 0.3%),-36 (− 3.1%)],[− 771 (− 66.6%), 21 (1.8%)], respectively.

**Conclusions:**

To date, the PEGeD algorithm is the most appropriate strategy among the authoritative algorithms for the likelihood prediction of pulmonary embolism in patients with AECOPD.

## Introduction

Chronic obstructive pulmonary disease (COPD)is a leading cause of morbidity and mortality worldwide that induces a substantially and increasingly economic and social burden [1, 2]. The acute exacerbation of chronic obstructive pulmonary disease (AECOPD) is an acute worsening of respiratory symptoms that results in additional therapy [3, 4].

Acute pulmonary embolism (PE) is the third most frequent acute cardiovascular syndrome behind myocardial infarction and stroke globally. Among patients who die of PE, 34% died suddenly before therapy could be initiated or take effect, 59% was diagnosed after death and only 7% were correctly diagnosed with PE before death [5]. PE that has an explicit indication for anticoagulant treatment is frequently encountered in AECOPD [6]. If it happens, PE is significantly associated with increased mortality and length of hospital stay in patients with AECOPD [7–9]. Consequently, PE should be ruled out when a patient with COPD has an acute exacerbation [10].

The hitherto globally recognized algorithms for the likelihood prediction of PE mainly comprise the Standard algorithm [11], the Age-adjusted algorithm [12], the YEARS algorithm [13], the PERC algorithm [14], and the PEGeD algorithm [15]. By the Standard algorithm, pulmonary embolism is ruled out by a D-dimer level of less than 500 nanogram(ng)/milliliter(ml) and a low clinical pretest probability (C-PTP) [11]. C-PTP is most frequently assessed via the Wells score [11] or the Geneva score [5]. By the Age-adjusted D-dimer algorithm, pulmonary embolism is ruled out in patients who are 50 years of age or younger and have a low or moderate C-PTP as well as a D-dimer level of less than 500 ng/ml, or who are older than 50 years of age with a D-dimer level that is less than 10 times the patient’s age [12]. By the YEARS algorithm, pulmonary embolism is ruled out in patients with none of the clinical signs of DVT, hemoptysis, or the most likely probability of pulmonary embolism and a D-dimer level of less than 1000 ng/ml and in those with one or more of the aforementioned three criteria and a D-dimer level of less than 500 ng/ml [13]. By the PERC algorithm, PE is ruled out in patients who meet all of the following criteria: age < 50 years, SaO2 > 94%, pulse < 100 beats per minute, no haemoptysis, no recent trauma or surgery, no history of VTE, no unilateral leg swelling, and no oral hormone use [14]. By the PEGeD algorithm, PE is ruled out in patients with a low C-PTP and a D-dimer level of less than 1000 ng/ml and in patients with a moderate C-PTP as well as a D-dimer level of less than 500 ng/ml [15]. For each algorithm, if PE cannot be ruled out while its likelihood being predicted by using the algorithm, the further chest imaging investigations for the confirmation of the presence or absence of PE are warranted. The summary of the variables being involved in each algorithm is in Table 1.

**Table 1:** The summary of the variables being involved in each algorithm

Variables	Standard	Age-adjusted	YEARS	PERC	PEGeD
DVT signs	+	+	+	+	+
PE likely	+	+	+	–	+
HR	+	+	–	+	+
Recent immobilization or surgery	+	+	–	+	+
History of VTE	+	+	–	+	+
Hemoptysis	+	+	+	+	+
Cancer	+	+	–	–	+
One D-dimer cutoff value	+	–	–	–	–
Two D-dimer cutoff values	–	+	+	–	+
Age	–	+	–	+	–
SaO2	–	–	–	+	–
Oral hormone use	–	–	–	+	–

+ denotes that the variable is involved in the algorithm; − denotes that the variable is not involved in the algorithm; *DVT* Deep Venous Thrombosis, *PE* Pulmonary Embolism, *HR* Heart Rate, *VTE* Venous Thromboembolism, *SaO2* Arterial Oxygen Saturation

In a previous study, the PEGeD algorithm and the YEARS algorithm were both regard as the safest strategies for PE prediction at the cost of minimum number of chest imaging performance in general population [15]. Nevertheless, for patients with AECOPD, which one of those algorithms has the best diagnostic accuracy to predict the likelihood of a PE? In other words, which algorithm can safely exclude PE for patients with AECOPD via the minimum number of chest imaging performance? Those questions had remained unanswered before the present study. Thus the current study was performed under such circumstances.

## Methods

### Study design

A retrospective study was conducted to investigate which one of the contemporarily authoritative algorithms for PE prediction had the best diagnostic accuracy for the prediction of PE in patients with AECOPD. We reviewed patients with AECOPD who had undergone computed tomography pulmonary angiography (CTPA) and/or planar ventilation/perfusion (V/Q) scan due to the suspected likelihood of PE which was assessed by the Standard algorithm during hospitalization. The C-PTP in the Standard algorithm was determined by using the Wells score then. In the current study, the patients’ likelihood of PE were reassessed via the Age-adjusted algorithm, the YEARS algorithm, the PEGeD algorithm, and the PERC algorithm, to compare their diagnostic accuracy for the likelihood prediction of PE. The Wells score value and D-dimer level had been adopted in the Standard algorithm then were adopted in the process of reassessment of PE by other algorithms which comprised the Wells score and D-dimer in the current study. All data was retrieved from the Electronic Medical Record (EMR) of three hospitals in Shanghai, including Shanghai Xinhua Hospital, Shanghai Pulmonary Hospital, and Shanghai Punan Hospital. This protocol was approved by the institutional review boards of the abovementioned hospitals.

### Study population

All eligible patients were collected according to the inclusion and exclusion criteria. The inclusion criteria comprised: 1) all eligible patients had a confirmed diagnosis of COPD according to the guidelines [16]; 2) all eligible patients with COPD had an acute exacerbation according to the guidelines [16]; 3) all eligible PE-suspected patients with AECOPD underwent CTPA and/or planar V/Q scan to confirm the presence or absence of PE. CTPA and V/Q scan were both performed if patients had no contraindication to the two examinations. The patients who were contraindicated to CTPA underwent V/Q scan only. According to the guidelines [5], PE was excluded if the results of both investigations were negative or the result of V/Q scan was negative when CTPA was not feasible, to ensure the true exclusion of PE. Meanwhile, PE was diagnosed if the results of both investigations were positive or either result of two investigations was positive. In patients with hemodynamic instability that was too critical to undergo CTPA or planar V/Q scan, bedside transthoracic echocardiogram or transoesophageal echocardiography were adopted to confirm the diagnosis. The exclusion criteria comprised: patients who had chronic pulmonary embolism.

### Statistical analyses

Measurement data were presented as mean ± standard deviation or median with interquartile range according to whether or not they were in normal distribution. Categorical data were presented as percentages. The comparison of measurement data between groups was performed by using T-test. The comparison of rates was performed by Chi-square test. The sensitivity, specificity, positive predictive value (PPV), negative predictive value (NPV), positive likelihood ratio (PLR), negative likelihood ratio (NLR), Youden index(YI), and diagnostic accuracy(DA) were compared among the Standard algorithm, the Age-adjusted algorithm, the YEARS algorithm, the PERC algorithm, and the PEGeD algorithm. The number of chest imaging examinations and missed diagnoses resulted from each algorithm were compared between every two diagnostic algorithms. SPSS 26 was used for the statistical analysis. Statistical significance was defined as a *P* value being less than 0.05.

## Results

### The demographics and characteristics of patients

According to the inclusion criteria, a total of 1288 eligible patients from Jan, 2015 through Dec, 2019 were retrieved from the EMR of the inpatient departments of three hospitals in Shanghai, China. After the exclusion of 130 patients with a medical history of chronic pulmonary embolism, 1158 patients entered the final analyses set. The demographic and clinical characteristic of patients were summarized in Table 2.

**Table 2:** The demographics and characteristics of patients

Variables	AECOPD(*n* = 948)	AECOPD-PE(*n* = 210)	*P* value
Age-years	66.9 ± 18.6	68.1 ± 20.3	0.858
Age<50-no.(%)	26(2.7)	5(2.4)	0.769
Age ≥ 50-no.(%)	922(97.3)	205(97.6)	
Female-no.(%)	303(32.0)	72(34.3)	0.515
Male-no.(%)	645(68.0)	138(65.7)	
Smoker-no.(%)	633(66.8)	145(69.0)	0.525
Nonsmoker-no.(%)	315(33.2)	65(31.0)	
BODE index	5.9 ± 3.6	6.2 ± 3.4	0.095
D-dimer-ng/ml	1191 ± 676	3118 ± 1635	<0.001
Wells score	3.3 ± 1.4	6.5 ± 3.3	<0.001
DVT sign-no.(%)	96(10.1)	66(31.4)	<0.001
No DVT sign-no.(%)	852(89.9)	144(68.6)	
PE likely-no.(%)	555(58.5)	177(84.3)	<0.001
PE unlikely-no.(%)	393(41.5)	33(15.7)	
HR ≤ 100beats/min-no.(%)	450(47.5)	102(48.6)	0.772
HR>100beats/min-no.(%)	498(52.5)	108(51.4)	
Recent immobilization-no.(%)	640(67.5)	186(88.6)	<0.001
No recent immobilization-no.(%)	308(32.5)	24(11.4)	
Recent surgery-no.(%)	99(10.4)	45(21.4)	<0.001
No recent surgery-no.(%)	849(89.6)	165(78.6)	
History of VTE	84(8.9)	21(10.0)	0.603
No history of VTE	864(91.1)	189(90.0)	
Hemoptysis-no.(%)	66(7.0)	12(5.7)	0.514
No hemoptysis-no.(%)	882(93.0)	198(94.3)	
Cancer-no.(%)	147(15.5)	54(25.7)	<0.001
No cancer-no.(%)	801(84.5)	156(74.3)	
SaO2 ≤ 94%-no.(%)	806(85.0)	188(89.5)	0.090
SaO2>94%-no.(%)	142(15.0)	22(10.5)	
Oral hormone use-no.(%)	275(29.0)	78(37.1)	0.021
No oral hormone use-no.(%)	673(71.0)	132(62.9)	

*AECOPD* Acute Exacerbation of Chronic Obstructive Pulmonary Disease, *PE* Pulmonary Embolism, *no.* number, *BODE* Body-Mass Index, Airflow Obstruction, Dyspnea, and Exercise Capacity, *DVT* Deep Venous Thrombosis, *HR* Heart Rate, *VTE* Venous Thromboembolism, *SaO2* Arterial Oxygen Saturation

Of a total of 1158 patients with AECOPD who underwent the decisive investigations of PE, the absence of PE was found in 948 patients, whereas the presence of PE was found in the remaining 210 ones. Among 210 AECOPD patients with confirmed PE, 8 critical patients were diagnosed with bedside transthoracic echocardiogram, 2 critical patients were diagnosed with transoesophageal echocardiography. Among the rest of 200 AECOPD patients with PE, 18 patients was diagnosed with PE via V/Q scan only due to the contraindication to CTPA. Among the rest of 182 patients, the results of CTPA was consistent with that of V/Q scan in 136 patients. Among the rest of 46 patients, 20 patients had positive results of CTPA and negative results of V/Q scan, whereas 26 patients had positive results of V/Q scan and negative results of CTPA. Among 948 patients whose PE diagnoses were ruled out, 864 patients had negative results of both CTPA and V/Q scan, whereas 84 patients whose PE diagnoses were excluded via V/Q scan only due to the contraindication to CTPA.

### The comparison of diagnostic accuracy for PE prediction among all algorithms

After the likelihood of PE of all eligible patients in the final analyses set was reassessed by the Age-adjusted algorithm, the YEARS algorithm, the PERC algorithm and the PEGeD algorithm, the results showed that the PEGeD algorithm(88.6, 80.7, 50.4, 97.0%, 4.591, 0.141, 0.693, 82.1%) performed better overall in the sensitivity, specificity, positive predictive value, negative predictive value, positive likelihood ratio, negative likelihood ratio, Youden index, and diagnostic accuracy, in comparison with the Age-adjusted algorithm (78.6, 74.1, 40.1, 94.0%, 3.034, 0.289, 0.527, 74.9%), the YEARS algorithm (71.4, 76.6, 40.3, 92.4%, 3.051, 0.373, 0.480,75.6%), the PERC algorithm (98.6, 1.6, 18.2, 83.3%, 1.002, 0.875, 0.002, 19.2%), and the Standard algorithm of which only PPV(18.1%) was available, since other variables were not applicable. The PERC algorithm, though having high sensitivity (98.6%), had very low specificity (1.6%). The sensitivity of the Age-adjusted algorithm (78.6%) was higher than that of the YEARS algorithm (71.4%), whereas its specificity (74.1%) was lower than that of the YEARS algorithm (76.6%). Although the Youden index of the Age-adjusted algorithm (0.527) was higher than that of the YEARS algorithm (0.480), its diagnostic accuracy (74.9%) was lower than that of the YEARS algorithm (75.6%). (Table 3).

**Table 3:** The comparison of the diagnostic accuracy for the likelihood prediction of PE among the Standard, the Age-adjusted, the YEARS, the PERC and the PEGeD algorithms for patients with AECOPD

Variables	Standard	Age-adjusted	YEARS	PERC	PEGeD
TP-no.	210	165	150	207	186
FP-no.	948	246	222	933	183
FN-no.	N/A	45	60	3	24
TN-no.	N/A	702	726	15	765
Sensitivity -%	N/A	78.6%	71.4%	98.6%	88.6%
Specificity -%	N/A	74.1%	76.6%	1.6%	80.7%
PPV -%	18.1%	40.1%	40.3%	18.2%	50.4%
NPV -%	N/A	94.0%	92.4%	83.3%	97.0%
PLR	N/A	3.034	3.051	1.002	4.591
NLR	N/A	0.289	0.373	0.875	0.141
YI	N/A	0.527	0.480	0.002	0.693
DA -%	N/A	74.9%	75.6%	19.2%	82.1%

*TP* True Positive, *FP* False Positive, *FN* False Negative, *N/A* Not Applicable, *TN* True Negative, *PPV* Positive Predictive Value, *NPV* Negative Predictive Value, *PLR* Positive Likelihood Ratio, *NLR* Negative Likelihood Ratio, *YI* Youden Index, *DA* Diagnostic Accuracy

### The difference of number of patients who were necessary to undergo chest imaging examinations and missed diagnoses resulted from each algorithm between every two diagnostic algorithms

The number of patients who were necessary to undergo chest imaging examinations by the Standard algorithm, the Age-adjusted algorithm, the YEARS algorithm, the PERC algorithm and the PEGeD algorithm were 1158, 411, 372, 1140 and 369, respectively. The number of missed diagnoses resulted from the Age-adjusted algorithm, the YEARS algorithm, the PERC algorithm and the PEGeD algorithm were 45, 60, 3, and 24, respectively. After the comparison, it was revealed that, the difference(absolute difference and its percentage of all 1158 patients)of number of patients who were necessary to undergo imaging examinations and missed diagnoses resulted from each algorithm between the PEGeD algorithm and the Standard algorithm, the Age-adjusted algorithm, the YEARS algorithm, as well as the PERC algorithm were [− 789 (− 68.1%), N/A], [− 42 (− 3.6%),-21 (− 1.8%)], [− 3 (− 0.3%),-36 (− 3.1%)],[− 771 (− 66.6%), 21 (1.8%)], respectively. Between the YEARS and the Age adjusted algorithms, it demonstrated that the YEARS algorithm led to less imaging examinations [− 39(− 3.4%)], whereas more missed diagnoses [15 (1.3%)], in comparison with the Age-adjusted algorithm. (Table 4).

**Table 4:** The difference of number of patients who were necessary to undergo chest imaging examinations and missed diagnoses resulted from each algorithm between every two diagnostic algorithms

Difference	Chest imaging-no. (%)	Missed diagnose-no. (%)
PEGeD-Standard	−789 (−68.1)	N/A
PEGeD-Age adjusted	−42 (−3.6)	−21 (−1.8)
PEGeD-YEARS	−3 (−0.3)	−36 (− 3.1)
PEGeD- PERC	−771 (−66.6)	21 (1.8)
YEARS-Standard	−786 (−67.9)	N/A
YEARS-Age adjusted	−39 (− 3.4)	15 (1.3)
YEARS-PERC	− 768 (− 66.3)	57 (4.9)
Age adjusted- Standard	− 747 (−64.5)	N/A
Age adjusted-PERC	− 729 (−63.0)	42 (3.6)
PERC-Standard	−18 (−1.6)	N/A

*N/A* Not Applicable

Taken together, among the contemporarily authoritative algorithms for the prediction of pulmonary embolism, the PEGeD algorithm had the best diagnostic accuracy for the prediction of PE in patients with AECOPD, followed by the YEARS algorithm and the Age-adjusted algorithm, while the PERC algorithm and the Standard algorithm underperformed in such a procedure.

## Discussion

The pretest prediction of pulmonary embolism is more critically vital for patients with AECOPD who have a higher risk of PE than the general population. The prevalence of PE in patients with AECOPD was approximately 16.1% [6], being close to the 18.1% (210/1158) in the current study, is well above that in general population which is approximately 0.1% [17]. It could be devastating for patients with AECOPD if their life-threatening pulmonary embolism are missed. However, having all patients with AECOPD tested for CTPA and/or V/Q scan will obviously waste plenty of medical resources and increase the suffering and adverse effects for the patients. As a result, to single out a prediction method which can highly accurately predict the likelihood of PE among the hitherto authoritative algorithms for the PE prediction is imperative for patients with AECOPD.

The variables involved in all five algorithms mainly comprise: DVT signs, PE likely, heart rate (HR), recent immobilization or surgery, history of VTE, hemoptysis, cancer, one D-dimer cutoff value(500 ng/ml), two D-dimer cutoff values(500 ng/ml, 1000 ng/ml or the age-adjusted), age, SaO2, and oral hormone use [11–15]. Among those variables, DVT signs and hemoptysis are both adopted in all five algorithms, whereas one D-dimer cutoff value is only adopted in the Standard algorithm, meanwhile, SaO2 and oral hormone use are both adopted in the PERC algorithm only. The PE likely, HR, recent immobilization or surgery, and history of VTE are all adopted in four algorithms, respectively. The two D-dimer cutoff values and cancer are both adopted in three algorithms, respectively. The number of variables contained in the Standard algorithm, the Age-adjusted algorithm, the YEARS algorithm, the PERC algorithm, and the PEGeD algorithm were 8, 9, 4, 8, and 8, respectively. (Table 1).

First of all, the results of the current study demonstrated that the Standard algorithm was not appropriate for the prediction of PE in patients with AECOPD, based on its poor PPV (18.1%) and the excessive number of chest imaging examinations compared with other algorithms, despite its most variables were unavailable due to the study design per se. The Standard algorithm which is widely used in clinical practice is a more cautious criterion for the PE screening, in comparison with the Age-adjusted algorithm, the YEARS algorithm, and the PEGeD algorithm. The probability of PE can only be excluded if both of low C-PTP and D-dimer level less than 500 ng/ml are met in the Standard algorithm [11]. However, due to the frequently higher D-dimer level [18] and more immobilization [19] in patients with AECOPD than those in general population, the Standard algorithm often leads to an increased false positive rate of PE prediction as well as an excessive imaging investigations instead of missed diagnoses.

Secondly, the same goes with the PERC algorithm. Being usually above 50 years old or tachycardic or hypoxic or oral glucocorticoid user are the common characteristics of patients with AECOPD, regardless of the suspicious degree of PE, whereas PE can be safely excluded only when all criteria of the PERC including age < 50 years, pulse < 100 beats per minute, SaO2 > 94%, and no oral hormone use are met by the PERC rule [14]. In this way the PERC rule makes almost every patient with AECOPD to be suspected to have a PE, which is obviously unreasonable. As a result, this algorithm also leads to a startling high false positive rate and excessive chest imaging investigations, although its sensitivity and number of missed diagnoses were 98.6% and 3, respectively.

Thirdly, this study suggested that the diagnostic accuracy of the Age-adjusted algorithm was slightly inferior to that of the YEARS algorithm, while their diagnostic accuracy were both inferior to that of the PEGeD algorithm. We think it may be because the Age-adjusted algorithm still applies the classic standard of D-dimer level to patients with AECOPD aged less than 50 years [12],which may lead to an increased false positive rate of PE prediction in that patient group. For the YEARS algorithm, in view of its C-PTP determination only measures three criteria in the Wells score, whereas omits important risk factors such as recent immobilization, recent surgery and cancer [13], accordingly the false negative rate may be elevated.

As a comparison, the PEGeD algorithm performed better in the Youden index, diagnostic accuracy, number of necessary imaging examinations, and number of missed diagnoses than all other algorithms, despite its sensitivity was lower than that of the PERC algorithm(difference, 10.0%) and its number of missed diagnoses was slightly more than that of the PERC(difference, 1.8%). We deem that maybe because it adopts the criteria consisted of the low C-PTP combined with the high D-dimer level (1000 ng/ml), the moderate C-PTP combined with the standard D-dimer level (500 ng/ml), and the high C-PTP without the reference to D-dimer [15], achieving the perfect complementarity between the C-PTP and D-dimer level, thus greatly improving the sensitivity and specificity and then the diagnostic accuracy of the prediction of PE likelihood in patients with AECOPD.

The implication of this study lies in that, for the first time, the most appropriate algorithm for the PE likelihood prediction in patients with AECOPD to date was discovered. The clinical and socioeconomic value of the PEGeD algorithm consists in that it can maximumly accurately identify the likelihood of PE in patients with AECOPD, so as to maximumly minimize the frequency of CTPA and V/Q scan and the missed diagnoses concurrently, thereby reducing the potentially physical injury resulted from imaging investigations, and avoiding unnecessary health care costs as well as the waste of medical resources, on the basis of ensuring medical safety.

This study has some limitations despite its values. First of all, it was a retrospective study. A similar prospective study is warranted in the future. Secondly, a few patients who were contraindicated to CTPA underwent V/Q scan only in the present study. Although it is recommended to reject the diagnosis of PE if the V/Q scan is normal, and to accept that the diagnosis of PE if the V/Q scan yields high probability for PE [5], the V/Q scan alone is slightly less convincing, compared with the combination of CTPA and V/Q scan in the diagnosis of pulmonary embolism.

## Conclusions

The results of the current study indicate that the PEGeD algorithm is the most appropriate authoritative strategy, compared with the Standard algorithm, the Age-adjusted algorithm, the YEARS algorithm and the PERC algorithm, for the likelihood prediction of pulmonary embolism in patients with AECOPD to date. The findings are expected to shed some light on the clinical practice in this field and be validated by prospective studies in the future.

## Data Availability

The datasets used and/or analysed during the current study are available from the corresponding author on reasonable request.

## Abbreviations

PE: Pulmonary Embolism
AECOPD: Acute Exacerbation of Chronic Obstructive Pulmonary Disease
COPD: Chronic Obstructive Pulmonary Disease
PERC: Pulmonary Embolism Rule-out Criteria
PEGeD: Pulmonary Embolism Graduated D-dimer
CTPA: Computed Tomography Pulmonary Angiography
V/Q: Ventilation/Perfusion
C-PTP: Clinical Pretest Probability
EMR: Electronic Medical Record
BODE: Body-Mass Index, Airflow Obstruction, Dyspnea, and Exercise Capacity
DVT: Deep Venous Thrombosis
HR: Heart Rate
VTE: Venous Thromboembolism
SaO2: Arterial Oxygen Saturation
TP: True Positive
FP: False Positive
FN: False Negative
TN: True Negative
PPV: Positive Predictive Value
NPV: Negative Predictive Value
PLR: Positive Likelihood Ratio
NLR: Negative Likelihood Ratio
YI: Youden Index
DA: Diagnostic Accuracy
